# Dry-Caribbean *Bacillus* spp. Strains Ameliorate Drought Stress in Maize by a Strain-Specific Antioxidant Response Modulation

**DOI:** 10.3390/microorganisms8060823

**Published:** 2020-05-30

**Authors:** Andres Moreno-Galván, Felipe A. Romero-Perdomo, German Estrada-Bonilla, Carlos Henrique Salvino Gadelha Meneses, Ruth R. Bonilla

**Affiliations:** 1 Corporación Colombiana de Investigación Agropecuaria—AGROSAVIA, Centro de Investigación Tibaitatá—Km 14 Vía Mosquera—Bogotá 250047, Cundinamarca, Colombia; aemoreno@agrosavia.co (A.M.-G.); fromerop@agrosavia.co (F.A.R.-P.); gaestrada@agrosavia.co (G.E.-B.); 2 Departamento de Biologia—Centro de Ciências Biológicas e da Saúde/Programa de Pós-Graduação em Ciências Agrárias, Universidade Estadual da Paraíba, Rua Baraúnas, 351, Bairro Universitário, Campina Grande-PB 58429-500, Brazil; chmeneses@gmail.com

**Keywords:** biochemical response, drought stress mitigation, plant growth-promoting bacteria (PGPB), *Zea mays*

## Abstract

Drought is a global problem for crop productivity. Therefore, the objective of this research was to evaluate five dry-Caribbean *Bacillus* spp. strains in drought stress amelioration in maize plants. Maize seeds were single-strain inoculated and sown in pots under greenhouse conditions. After 12 days, plants were subjected to 33 days of drought conditions, i.e., 30% of soil field capacity, and then collected to measure leaf and root dry biomass, plant height, antioxidant enzymes, proline accumulation, and P^+^, Ca^2+^, and K^+^ uptake. Results correlated drought stress amelioration with the inoculation of *Bacillus* spp. strains XT13, XT38 and XT110. Inoculated plants showed increases in dry biomass, plant height, and K^+^ and P^+^ uptake. The overall maize antioxidant response to bacterial inoculation under drought stress showed dependence on proline accumulation and decreases in ascorbate peroxidase and glutathione reductase activities. Moreover, results suggest that this stress amelioration is driven by a specific plant-strain correlation observed in antioxidant response changes in inoculated plants under stress. Also, there is a complex integration of several mechanisms, including plant growth-promotion traits and nutrient uptake. Hence, the use of dry-Caribbean plant growth-promoting *Bacillus* strains represents an important biotechnological approach to enhance crop productivity in arid and semi-arid environments.

## 1. Introduction

Drought is a limitation for crop productivity with economic, social, and environmental impacts at the global level [1,2,3]. Furthermore, climate change is increasing the severity, duration, and frequency of drought in several crops used for human and animal feeding [4]. This water deficit leads to the reduction of plant growth by the production of reactive oxygen species (ROS), reducing photosynthetic activity, membrane stability, and inducing irreversible damage to biological molecules that finally trigger cell death [5,6].

Plants use enzymatic and non-enzymatic antioxidant mechanisms to cope with the deleterious effects of ROS. The antioxidant enzymes have two main roles, one is focused towards ROS detoxification, such as ascorbate peroxidase (APX), and the other role is related to the restoration of antioxidant molecules like glutathione reductase (GR) [7,8]. The APX enzyme uses ascorbic acid to transform the hydrogen peroxide into water, controlling the ROS concentration; meanwhile, the GR enzyme maintains the redox balance restoring reduced glutathione (GSH), and thus helps other enzymes to restore the ascorbate used by the APX [9].

The non-enzymatic antioxidants are mainly low weight molecules that are accumulated by the plants to maintain cellular water homeostasis, such as proline and some sugars. These molecules reduce ROS damage by preventing its interaction with plant key molecules like DNA, proteins, and lipids [8,10].

The overall effect of plant growth-promoting bacteria (PGPB) inoculation in drought stress amelioration is related to reducing plant biomass losses associated with the ROS effects [11,12,13]. PGPB inoculation has been shown to ameliorate abiotic stresses in many plants, including *Zea mays* (maize) and other grasses [5,14,15,16]. There are several reports of inoculation with *Bacillus* spp. strains that induce drought amelioration in plants [17,18,19]; this occurs due to plant growth promotion through phytohormone synthesis, production of volatile compounds, and increase of nutrient availability [17,20,21].

The *Bacillus* genus is one of the most common and important isolated rhizobacteria due to the wide range of functionalities and their ability as biological stimulants [2,12,21]. The genus includes several species with biotechnological applications from bioremediation, pathogen control, and enzymes and antibiotic production, to probiotics and other biochemicals [12,21], but their adaptation and survival to several environmental conditions make them an outstanding PGPB for stimulating plant growth and increasing crop yield [2,3,15,18,21].

In a previous study, a *Bacillus* spp. consortium modulated *Megathyrsus maximus* (Guinea grass) antioxidant response leading to drought stress amelioration through biomass production increase [15]. These strains may lead to the development of biotechnologies to increase crop production in arid and semiarid regions due to their ability to endure harsh environments and mitigate plant drought stress. Thus, a better comprehension of their single effect in the antioxidant modulation of a plant is needed to gain insights into their possible cooperative work in drought-stress mitigation. Accordingly, the objective of this work was to study the effect of the single inoculation of five Dry-Caribbean *Bacillus* spp. strains on the growth and antioxidant response of a drought-sensitive plant like maize under water deficit.

## 2. Materials and Methods

### 2.1. Strains, Phylogenetic Analysis and Culture Conditions

Five strains of *Bacillus* spp. previously isolated from the Dry-Caribbean region of Colombia [15] were screened for their ability to ameliorate drought stress in maize plants in this study. The 16S rRNA gene was amplified by PCR and sequenced as previously described [15]. The almost complete 16S rRNA genes of strains XT13 (1344 bp), XT14 (1266 bp), XT38 (905 bp), and XT110 (1328 bp) were then aligned with those of the type material strains of the genera belonging to the order *Bacillales* obtained from the NCBI Reference RNA Sequences database. The strains sequences of 16S rRNA were deposited previously in NCBI by [15]. The accession numbers of the strains were XT13—MH014814; XT14—MH014816; XT17—MH014818; XT38—MH014817; and XT110—MH014815. The phylogenetic dendrograms were reconstructed using the Neighbor-Joining method using the Tamura-Nei genetic distance model based on a Geneious Aligment—global alignment with free end gaps and a cost matrix of 93% similarity (5.0/−9.026168) included in the Geneious Tree Builder Tool of Geneious Prime 2020.1.2 (https://www.geneious.com) software.

All strains were cultured in Tryptic Soy Agar (TSA) (Merck KGaA, Darmstadt, Germany) and incubated for 24 h at 30 °C ± 2 °C. Liquid cultures of these strains were obtained from a single colony inoculated in Tryptic Soy Broth (TSB) (Merck KGaA, Darmstadt, Germany) and incubated for 24 h at 30 °C ± 2 °C and exposed to orbital shaking at 150 rpm.

### 2.2. Plant Growth-Promoting (PGP) Traits

The indole-like compounds were estimated using the colorimetric assay based on the Salkowsky PC reagent [22]. Flasks with 5 mL of TSB and 5 mM of L-tryptophan were inoculated with 100 µL of a standardized suspension of each strain (optical density of 0.5 at 600 nm) and incubated for 48 h in darkness at 30 °C ± 2 °C and exposed to orbital shaking at 150 rpm. The reaction and determination of indole-like compounds were performed according to [23].

The phosphate-solubilizing activity in NBRIP liquid medium was measured according to [24], soluble phosphate amounts were quantified by the phosphomolybdate blue method at 712 nm [25].

Phytate hydrolyzation activity was measured as described by [26] using the sodium phytate medium approach with modifications. A solid medium was prepared by adding 14 g L^−1^ agar-agar (Merk KGaA, Darmstadt, Germany) to the reported sodium phytate medium. The medium was inoculated using three drops, each of 50 µL of a standardized suspension (optical density of 0.5 at 600 nm) per strain in 8.5 g L^−1^ NaCl and incubated five days at 30 °C ± 2 °C. After incubation, positive results were associated with the appearance of clear zones around the bacterial drops. 

ACC-deaminase activity was measured by monitoring the amount of α-ketobutyrate produced due to ACC hydrolysis carried out by the rhizobacterial isolates containing ACC-deaminase, as described by [27].

The production of extracellular polymeric substances (EPS) by the strains was evaluated using the method described by [28] with modifications. From a TSB liquid culture of each strain, a 9.75 mL aliquot was supplemented with 0.4 mL of 0.26 M EDTA and 0.2 mL of 5 M NaCl and centrifuged at 5220× *g* for 10 min. The supernatant obtained was mixed with 10 mL of cold absolute ethanol (−86 °C), vigorously agitated, and centrifuged again. The supernatant was discarded, and the EPS pellet was reconstituted in 10 mL of deionized water. The EPS productions were determined by the quantification of total carbohydrates using the colorimetric phenol sulfuric method [29] measuring the absorbance at 490 nm and comparing against a glucose standard curve.

The production of the hydrolytic enzymes α-amylase, cellulase, pectinase, and protease by the strains were measured using the methods described by [30].

### 2.3. Effect of Drought and Bacillus spp. Inoculation on Maize Plant Growth

To study the effect of the inoculation of *Bacillus* spp. strains on drought stress amelioration in maize plants, seven treatments were designed based on individual inoculations of the strains, including watered and drought controls (Table 1). Greenhouse experiments were conducted in a completely randomized block design with five replicates per treatment under semi-controlled conditions.

The maize V-508 seeds (Semicol S.A.S, Bogotá, Colombia) were surface sterilized by soaking in 5% (*v*/*v*) sodium hypochlorite for 3 min, then with 70% (*v*/*v*) ethanol for 2 min and rinsed three times with sterilized water. One colony of each reactivated strain (Section 2.1) was inoculated in TSB medium and incubated for 24 h at 30 °C ± 2 °C and subjected to orbital shaking at 150 rpm. After incubation, CFU mL^−1^ content was measured in TSA, and the cell suspension was adjusted to ≈1 × 10^8^ CFU mL^−1^ with 8.5 g L^−1^ of NaCl solution. Seed inoculation was performed by dipping sterilized seeds for 2 h and exposed to orbital shaking at 200 rpm in the previously standardized cell suspension of each strain.

The pots were filled with 1.5 kg of clay loam soil previously sieved (2 mm/10-mesh), autoclaved for 1 h, and analyzed for physicochemical characteristics (Table 2). The soil was amended for maize requirements before filling the pots with 183.33 mg kg^−1^ of urea, 6.66 mg kg^−1^ of MgSO_4_, and 13.33 mg kg^−1^ of ZnSO_4_.

The soil was set to 70% of field capacity, and three seeds were sown per pot, leaving only one plant per pot after 12 days of germination. Plants were kept at 30% of field capacity until they reached 45 days, and then plant height, dry leaf, root biomass, proline accumulation, and ascorbate peroxidase and glutathione reductase activities were measured.

To score the ability of the dry-Caribbean *Bacillus* spp. strains to induce drought-stress amelioration in maize plants, a stress alleviation factor (SAF) as defined by [31] integrating plant height, and shoot and root biomass were calculated. The SAF was calculated for each strain using Equation (1):

(1) $$\mathrm{SAF}=\frac{\left(\left(\left(\frac{Ih-Ch}{Ch}\right)\times 100\right)+\left(\left(\frac{Is-Cs}{Cs}\right)\times 100\right)+\left(\left(\frac{Ir-Cr}{Cr}\right)\times 100\right)\right)}{3}$$

where: *I* = *Bacillus* sp. treatment; *C* = control without inoculation subjected to drought stress; *h* = plant height; *r* = root dry biomass; *s* = shoot dry biomass.

#### 2.3.1. Antioxidant Enzymatic Activities and Proline Content in Plant Tissue

Plant extracts were obtained by grinding the leaf tissue with liquid nitrogen in a cold mortar. A sample of 100 mg of the ground tissue was mixed with 2 mL of 0.2 M phosphate buffer (pH 7.8) containing 0.1 mM of EDTA in 2.5-mL microtubes and softly homogenized with a vortex. The homogenate was centrifuged at 15,000× *g* for 20 min at 4 °C (Eppendorf 5415C Centrifuge, Hamburg, Germany), and the supernatant was used to establish the enzymatic activities [15,32].

The APX activity (EC 1.11.1.11) was measured from the decrease in absorbance at 290 nm in 3 min using the method described by [33] and following the procedure described by [15]. The extinction coefficient for reduced ascorbate (2.8 mM^−1^ cm^−1^) was used to calculate the enzymatic activity per gram of fresh weight.

The GR activity (EC 1.20.4.2.) was measured from the increase in absorbance at 412 nm in 3 min, according to [34] and following the procedure described by [15]. The extinction coefficient of 5-thio-nitrobenzoic acid (TNB) (14.15 M^−1^ cm^−1^) was used to calculate the enzymatic activity per gram of fresh weight per minute.

The proline content in maize leaves was measured using the acid ninhydrin method reported by [35] following the procedure described by [15]. The calibration curve was generated using pure L-proline (Merck KGaA, Darmstadt, Germany) as the standard reference.

#### 2.3.2. Determination of P^+^, K^+^, and Ca^2+^ in Plant Tissue

Shoot samples for each treatment were pooled and divided in three, then washed several times with deionized water and oven-dried at 60 °C for 48 h. Samples were grounded, and 200 mg of each plant tissue was digested with an acid mixture following the procedure described by [23]. An Absorption Atomic Spectrophotometer (AAS 2380, Perkin Elmer, Waltham, MA, USA) was used for measuring the concentration of P^+^, K^+^, and Ca^2+^.

### 2.4. Statistical Analysis

The data were subjected to one-way statistical analysis of variance (ANOVA), followed by Tukey’s test. Differences were considered significant at *p* < 0.05. All statistical analyses were performed using the statistical software IBM SPSS version 22 (IBM Corp., Armonk, NY, USA). Pearson’s correlation coefficient was determined using the R software (R Development Core Team).

## 3. Results

### 3.1. Strains Characterization and Plant Growth-Promoting (PGP) Traits

**Figure 1:**
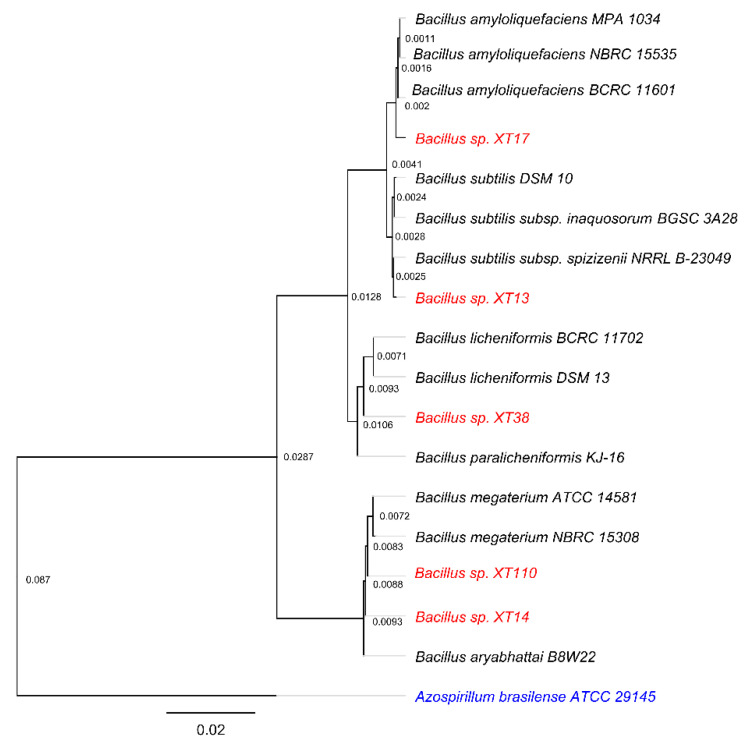
Phylogenetic dendrogram based on a comparison of the 16S rRNA gene sequence of strains XT13, XT14, XT17, XT38, and XT110, using type strains of the genera in the order *Bacillales*. The tree was created using the Neighbor-Joining method and the Tamura-Nei genetic distance model. The strains used in this study are indicated in red. The *Azospirillum brasilense* ATCC 29145 sequence was used as root for the tree and is indicated in blue. Scale bar, 0.2 nucleotide substitution per 10 nucleotides.

Phylogenetic analyses of the 16S rRNA gene sequence of strains XT13 (1344 bp), XT14 (1266 bp), XT38 (905 bp), and XT110 (1328 bp) with those of the type species of the genera belonging to the order *Bacillales* indicated that these strains belonged to the *Bacillus* genus and different species. According to the neighbor-joining and maximum-likelihood phylogenetic tree, the closest relatives to strain XT13 were *Bacillus subtilis* subsp. spizizenii NRRL B-23049 (99.78% identity), *Bacillus subtilis* subsp. inaquosorum (99.70%) and *Bacillus subtilis* DSM 10 (99.70%). The closest relatives to strain XT14 were *Bacillus aryabhattai* B8W22 (100%), *Bacillus megaterium* NBRC 15308 (99.85%), and *Bacillus megaterium* ATCC 14581 (99.85%). The closest relatives to strain XT17 were *Bacillus amyloliquefaciens* MPA 1034 (99.76%), *Bacillus amyloliquefaciens* NBRC 15535 (99.76%), and *Bacillus amyloliquefaciens* BCRC 11601 (99.68%). The closest relatives to strain XT38 were *Bacillus licheniformis* BCRC 11702 (100%), *Bacillus licheniformis* DSM 13 (100%), and *Bacillus paralicheniformis* KJ-16 (100%). The closest relatives to strain XT110 were *Bacillus aryabhattai* B8W22 (100%), *Bacillus megaterium* NBRC 15308 (99.84%), and *Bacillus megaterium* ATCC 14581 (99.84%) (Figure 1).

All strains were able to synthesize indole-like molecules using tryptophan as a precursor, solubilize Ca_3_PO_4_, hydrolyzate Phytate, and produce EPS (Table 3). The *Bacillus* sp. strains XT17 and XT110 produced the highest amounts of indole-like molecules; meanwhile, *Bacillus* sp. XT110 showed a remarkable activity for phytate hydrolyzation as well as Ca_3_PO_4_ solubilization. *Bacillus* sp. XT14 and XT38 showed the most significant EPS production. Among the hydrolytic enzyme activities, protease was the one that was observed most frequently among the strains. Nonetheless, cellulase, pectinase, and amylase activities were also observed in equal proportions between the strains (Table 3). *Bacillus* sp. XT17 exhibited all the hydrolytic enzyme activities, followed by *Bacillus* sp. XT13 and XT38. None of the isolates displayed ACC-deaminase activity under the evaluated conditions.

### 3.2. Bacillus spp. Inoculation Promotes Maize Growth and Nutrient Uptake under Drought Stress

**Figure 2:**
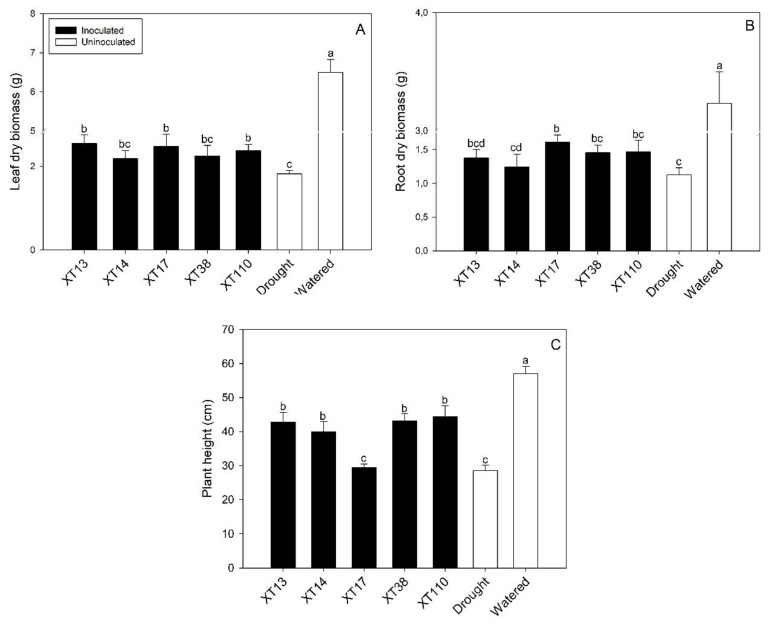
Effect of drought and *Bacillus* spp. inoculation on maize plants expressed as: (**A**) leaf dry biomass, (**B**) root dry biomass, and (**C**) plant height. Whiskers represent the standard deviation. Bars followed by the same letter are not significantly different at *p* ≤ 0.05 by Tukey’s test.

Plant growth decreased significantly under drought stress; reductions in plant height (50.0%) and root (65.2%) and leaf (72.0%) dry biomass were observed in uninoculated plants (Figure 2A–C). Moreover, PGPB inoculation was observed to increase total biomass in plants under drought stress. The inoculation with *Bacillus* sp. XT110 (31.0%), XT17 (36.0%), and XT13 (40.0%) increased leaf dry biomass on stressed plants when compared to the uninoculated stressed control (Figure 2A). Also, plants inoculated with *Bacillus* sp. XT17 showed the highest significant increase of dry root biomass under drought conditions (43.5%), followed by XT38 (30.4%) and XT110 (29.4%) when compared with the uninoculated drought control (Figure 2B). Plant height increased significantly by inoculation with *Bacillus* sp. XT13 (50.2%), XT14 (40.1%), XT38 (51.1%), and XT110 (55.8%) when compared with the uninoculated drought control (Figure 2C).

The SAF scores obtained showed higher values in treatments with *Bacillus* sp. XT110 (39.04), XT13 (37.48), and XT38 (34.60). Meanwhile, the strains XT17 (27.65) and XT14 (23.58) showed lower SAF scores. The uninoculated drought and watered controls scored 0.00 and 187.74, respectively.

*Bacillus* spp. inoculation increased K^+^ over P^+^ uptake in maize plants under drought stress (Table 4). Plants treated with *Bacillus* sp. XT14 recorded the largest K^+^ accumulation (22.5%), followed by XT38 (17.1%), XT17 (14.6%), XT13 (12.3%) and XT110 (11.8%) when compared with the uninoculated drought control. Furthermore, P^+^ accumulation increased significantly in stressed plants treated with *Bacillus* sp. XT14 (34.9%) and XT110 (42.8%) when compared with the uninoculated drought control. Further, the inoculation with *Bacillus* sp. XT13 significantly decreased Ca^2+^ (15.9%) in plant tissue when compared to the uninoculated drought control (Table 4).

### 3.3. Bacillus spp. Differentially Modulates Antioxidant Response in Maize Inducing Drought Stress Mitigation

**Figure 3:**
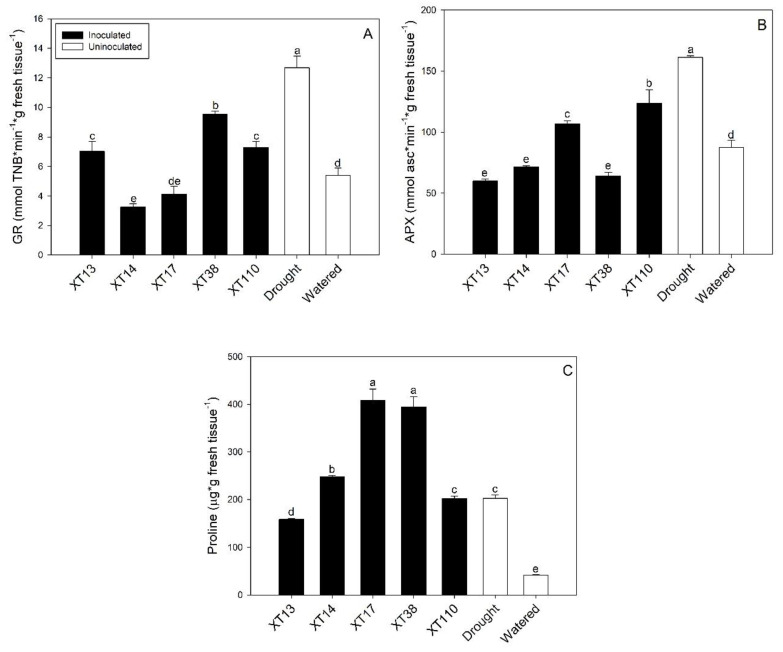
Effect of drought and *Bacillus* spp. inoculation on maize plants expressed as: (**A**) glutathione reductase (GR) activity, (**B**) ascorbate peroxidase (APX) activity, and (**C**) proline accumulation. Whiskers represent the standard deviation. Bars followed by the same letter are not significantly different at *p* ≤ 0.05 by Tukey’s test.

The antioxidant response to drought stress was stimulated in uninoculated plants, increasing GR (134.3%) and APX (84.7%) activities, as well as proline accumulation (387.17%) (Figure 3A–C). Moreover, *Bacillus* spp. inoculation showed to mitigate the plant enzymatic antioxidant responses under drought stress. The higher reductions in GR activity were recorded in plants inoculated with *Bacillus* sp. XT14 (74.4%) and XT17 (67.5%) (Figure 3A) when compared to the uninoculated drought control. Also, a significant reduction in APX activity was observed in plants inoculated with *Bacillus* sp. XT13 (62.2%), XT14 (55.6%), and XT38 (60.3%) (Figure 3B) when compared to the drought control. Proline accumulation increased significantly in drought-stressed plants treated with *Bacillus* sp. XT14 (22.1%), XT17 (101.7%), and XT38 (94.8%); in contrast, plants inoculated with *Bacillus* sp. XT13 showed a decrease of 21.8% when compared to the uninoculated drought control (Figure 3C).

### 3.4. Correlation between Plant Growth and Antioxidant Response with Bacillus Inoculation under Drought Stress

**Figure 4:**
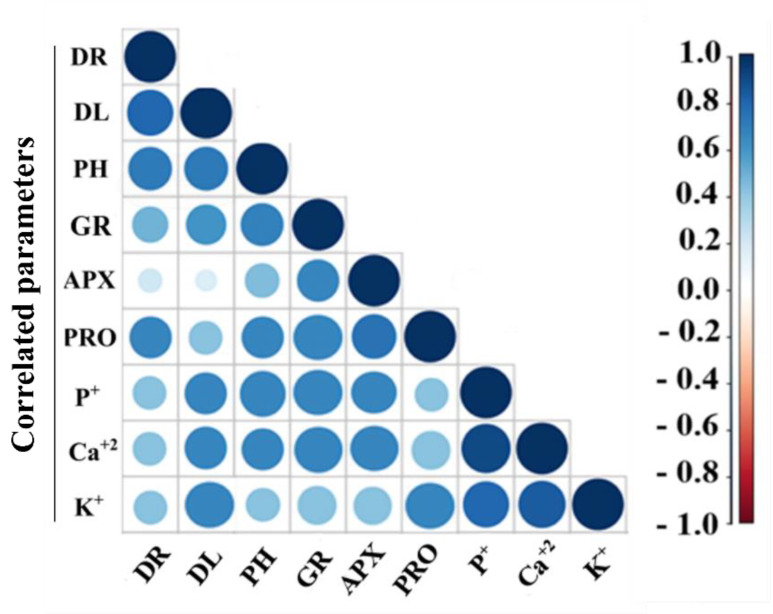
Pearson’s correlation matrix between plant growth, antioxidant enzymes, proline accumulation and nutrient uptake in plants inoculated with *Bacillus* spp. after 33 days of drought stress. Correlations are displayed in blue (positive) and red (negative); color intensity and circle size are proportional to the correlation coefficient. DR: dry root biomass, DL: dry leaf biomass, PH: plant height, GR: glutathione reductase activity, APX: ascorbate peroxidase activity, PRO: proline accumulation in plant tissue, P^+^: phosphorous concentration in plant tissue, Ca^+2^: calcium concentration in plant tissue, and K^+^: potassium concentration in plant tissue.

We observed a strong correlation between dry root biomass (DR), dry leaf biomass (DL) and plant height (PH), being this associated with an overall plant growth induced by *Bacillus* sp. inoculation under drought stress conditions. PH and DL showed a strong correlation with GR and proline accumulation in inoculated plants under drought stress. Dry leaf biomass showed to be correlated with an overall nutrient uptake (P^+^, Ca^+2^ and K^+^). Plant antioxidant responses (APX, GR and proline accumulation) showed a strong correlation between them. Also, GR and APX activities showed to be correlated with Ca^+2^ and P^+^ uptake in leaf tissue. In the other hand, proline showed to be correlated with K^+^ uptake in leaf tissue. Nutrient uptake of P^+^, Ca^+2^ and K^+^ showed a strong correlation between them. Also, Ca^+2^ and P^+^ uptakes were correlated with APX and GR activities as well as with PH and DL gains, but not with proline accumulation on *Bacillus* sp. inoculated plants under drought stress (Figure 4).

## 4. Discussion

In this study, the inoculation of maize plants with dry-Caribbean *Bacillus* spp. strains ameliorated drought stress effects in these plants. The inoculated plants under drought stress showed increases in nutrient uptake, plant height, root, and leaf biomass, as well as the downregulation of the GR and APX activities and changes in proline accumulation. All these effects have been closely related with drought stress amelioration by PGPB in maize [2,36], wheat [6] chickpea [37] and Guinea grass [15,38]. 

The inoculation of almost all the dry-Caribbean *Bacillus* spp. strains in the current study showed a consistent drought stress amelioration effect in maize plants, agreeing with the work of [15] in Guinea grass, despite the differences in the intensity and duration of the water deficit applied. Interestingly, we could not associate drought amelioration to an overall effect in the measured antioxidant variables, as each strain caused different antioxidant responses on maize under drought stress.

For instance, the *Bacillus* sp. strains XT13, XT38, and XT110 showed an interesting amelioration of the effects related to plant growth under drought stress according to the SAF score but reached by different biochemical antioxidant mechanisms. The *Bacillus* sp. XT110 treatment obtained the highest SAF score and showed a higher downregulation of the GR activity (−42.6%), together with a lower reduction of the APX activity (−23.4%), but with no changes in proline accumulation. In contrast, the *Bacillus* sp. XT38 treatment revealed a notable SAF score showing a lower reduction in GR activity (−24.8%), while a higher reduction in APX activity (−60.3%) was accompanied by a strong promotion of proline accumulation (94.8%). Interestingly, the *Bacillus* sp. XT13 treatment with the last remarkable SAF score showed strong downregulation for both GR (−44.6%) and APX (−62.8%) activities but accompanied by a reduction in proline accumulation (−21.8%). Similar results were obtained by [39] where single inoculated *Bacillus* sp. maize plants under drought stress showed proline increases ranging from 46% to 80%. In the same work, all inoculated plants decreased APX activity when compared to drought control but showed different values not only for APX, but for glutathione peroxidase (GPX) and catalase (CAT) enzymes. Interestingly, they did not discuss the correlation between antioxidant response modulation and strain inoculation.

Our findings suggest that despite the effect in biomass production under drought stress, the mechanisms involved in this amelioration depend on the *Bacillus* sp. strains used and their specific plant-bacterium relationship.

In the case of nutrient uptake, all the plants inoculated with *Bacillus* spp. showed an enhanced K^+^ accumulation under drought stress, finding a positive correlation between this accumulation and the effect on leaf biomass and proline on inoculated plants under drought stress (Figure 4). A similar effect was observed by [40] where the inoculation of *Bacillus* spp. ameliorated drought stress in *Lavandula* and *Salvia* by controlling proline and K^+^ accumulation. Potassium works as an inorganic osmolyte maintaining water homeostasis, being able to regulate stomatal opening, osmotic balance, maintenance of turgor pressure, and reduction of transpiration [12,41].

The P^+^ uptake showed an interesting behavior in plants inoculated with the *Bacillus* sp. strains XT14, and XT110. These strains showed higher in vitro values for both inorganic and organic phosphate solubilization, and plants inoculated with the strains showed the highest P^+^ accumulation under drought stress. Phosphorous availability plays an important role in plant response to drought stress as observed in cowpea [42] and bamboo [43] where P^+^ supplementation successfully ameliorated drought stress. Our findings showed that the increased uptake of P^+^ by inoculated plants under drought stress was correlated with antioxidant enzymatic activity and leaf biomass gains supporting its role in stress amelioration (Figure 4).

Increases of biomass and nutrient uptakes under drought stress may be explained through PGP-traits exhibited by the *Bacillus* spp. strains used in this study. The in vitro EPS production observed in all strains could be related with bacterial protection and plant colonization during drought stress; according to [44] the accumulation of EPS protected the cells of *Gluconoacetobacter diazotrophicus* against oxidative stress under in-vitro conditions and during the colonization of rice plants. Moreover, roots secrete organic compounds used by the bacteria for phytohormone biosynthesis; these compounds promote plant-cell division rate in roots and shoots, and, hence, increasing biomass gains and nutrient uptake [45,46,47].

In this study, uninoculated plants under water deficit showed that their antioxidant response to drought stress relied on increasing proline accumulation and GR activity. This behavior was observed in other drought stress studies in maize [6,48,49], as well as in other plants like Guinea grass [15]. These mechanisms seem to play an essential role in the maize antioxidant response under drought stress. 

In relation to the previous, almost all drought-stressed plants inoculated with *Bacillus* spp. showed to promote proline accumulation. Therefore, proline seems to be related to root biomass increases, antioxidant enzymes activity, and K^+^ uptake on inoculated plants (Figure 4). Proline is a potent antioxidant and a potential inhibitor of programmed cell death; it stabilizes and protects membranes, proteins, and DNA, and scavenge ROS to maintain osmotic adjustment [11,50,51,52]. Several bacteria have shown to stimulate proline production in plants under drought stress, hence promoting drought amelioration [4,8,53]. Interestingly, plants inoculated with *Bacillus* sp. XT13 showed a decrease in proline accumulation (after a 33-day drought stress) and exhibited drought stress amelioration. This could be related with an early proline accumulation as observed by [38] where rice plants inoculated with *Bacillus* sp. showed proline increases after 3 h under drought stress. In relation, *Bacillus* sp. co-inoculation in Guinea grass induced an early proline accumulation after 48 h of drought stress [15]. Therefore, reaching the necessary proline concentration in combination with the other modulated antioxidant responses could be enough for *Bacillus* sp. XT13 to ameliorate drought stress.

Our findings showed that *Bacillus* spp. inoculation reduces GR and APX activities in maize under drought stress. On the other hand, the correlation analysis showed a relation between leaf biomass increase and GR activity, but not with APX activity (Figure 4). This may suggest that savings in reduced glutathione (GSH) associated with the reduction in GR activity are also related to NADPH saving, which is essential in GSH enzymatic restoration [51,54]. The NADPH saved could be redirected towards carbon fixation in Calvin’s cycle, explaining in part, the increases in leaf biomass [55]. Besides, both enzymatic activities seem to be correlated with P^+^ uptake on stressed inoculated plants that were also correlated with leaf biomass gains on inoculated plants (Figure 4).

The *Bacillus* genera have shown to be a useful ally in the amelioration of drought and other abiotic stresses in several crops, thus, modulating similar responses consistent with our results [17,18,19,45].

Our previous study in Guinea grass [15] showed the drought stress amelioration ability of the co-inoculation of five *Bacillus* spp. strains (used in the current study) under non-sterile soil conditions. Nonetheless, our work in maize suggests that these five dry-Caribbean *Bacillus* spp. strains individually induce a similar drought stress amelioration effect in plant growth under sterile soil conditions, which depends on a specific plant-strain interaction observed in the differences in the evaluated antioxidant responses.

## 5. Conclusions

This study demonstrates the positive effect of *Bacillus* spp. inoculation on drought stress effects in maize. Nevertheless, this amelioration is driven by a specific plant-strain interaction at the species level that deferentially modifies antioxidant responses in the plant. Also, other mechanisms are integrally involved in this complex interaction, including the increase in P^+^ and K^+^ uptake, as well as plant-growth promotion abilities of the strains.

These Dry-Caribbean *Bacillus* sp. strains have an attractive biotechnological potential for arid and semi-arid regions relying on both their soil drought adaptation and their individual host-specific interaction with a consistent effect in growth promotion under drought stress in crops for human or livestock feeding like maize and Guinea grass.

## Figures and Tables

**Table 1:** Description of the treatments used to evaluate the effect of plant growth-promoting bacteria (PGPB) inoculation on the response of maize to drought stress.

Treatment	Description	Drought (Days) ^1^
Watered	Uninoculated control	None
Drought	Uninoculated control	33
XT13	Seeds inoculated with the XT13 strain	33
XT14	Seeds inoculated with the XT14 strain	33
XT17	Seeds inoculated with the XT17 strain	33
XT38	Seeds inoculated with the XT38 strain	33
XT110	Seeds inoculated with the XT110 strain	33

^1^ Drought as water deficit for 33 days at 30% field capacity. None: soil at full field capacity.

**Table 2:** Physicochemical soil characteristics used in the plant growth promotion experiment.

pH	O.M	Ca	Mg	K	Na	C.E.C	Fe	Mn	Zn	Cu	P	S	B
	%	cmol_(+)_ kg^−1^					mg kg^−1^						
6.97	2.50	7.04	0.86	1.42	0.02	9.34	51.60	3.80	2.50	2.20	347.15	4.10	0.34

O.M: organic matter, C.E.C: Cationic exchange capacity.

**Table 3:** Plant growth-promoting (PGP) characteristics of the *Bacillus* spp. strains assessed.

Characteristics	XT13	XT14	XT17	XT38	XT110
*PGP-features*					
ILM	0.65 ± 0.00	0.64 ± 0.00	0.65 ± 0.00	0.64 ± 0.01	0.66 ± 0.00
Ca_3_PO_4_	0.19 ± 0.01	0.23 ± 0.03	0.20 ± 0.02	0.19 ± 0.00	0.32 ± 0.02
Phytate	2.83 ± 0.44	4.51 ± 0.78	2.82 ± 0.54	1.13 ± 0.17	6.31 ± 0.24
EPS	0.06 ± 0.01	0.14 ± 0.00	0.07 ± 0.00	0.18 ± 0.00	0.07 ± 0.00
ACCd	N.D.	N.D.	N.D.	N.D.	N.D.
*Activities of hydrolytic enzymes*					
Protease	+	+	+	−	+
Cellulase	−	−	+	+	−
Pectinase	+	−	+	−	−
α-amylase	−	−	+	+	-

XT13: *Bacillus subtilis*; XT14: *Bacillus aryabhattai*; XT17: *Bacillus amyloliquefaciens*; XT38: *Bacillus licheniformis*; and XT110: *Bacillus aryabhattai*. ILM: indole-like molecules production in mg mg protein^−1^. Ca_3_PO_4_ solubilization as mg (PO_4_)^3−^ mg protein^−1^. Phytate: hydrolyzation as a clear zone in mm. EPS: exopolysaccharides production as mg glucose mg protein^−1^. ACCd: ACC-deaminase activity (µmoles α-KB h^−1^.mg protein^−1^). N.D: not detected. For hydrolytic enzymes activities, + indicates a positive reaction, while − indicates a negative reaction.

**Table 4:** Influence of drought stress and *Bacillus* spp. inoculation on leaf uptake of P^+^, K^+^, and Ca^2+^ in maize plants.

Treatment	P^+^	Ca^2+^	K^+^
	mg g^−1^ Dry Tissue		
XT13	3.27 ± 0.38	6.50 ± 0.17 *	46.03 ± 1.33 *
XT14	3.73 ± 0.06 *	8.30 ± 0.27	50.23 ± 0.81 *
XT17	3.20 ± 0.27	6.80 ± 0.20	46.97 ± 0.21 *
XT38	3.23 ± 0.21	8.30 ± 0.61	48.00 ± 2.01 *
XT110	3.95 ± 0.25 *	8.40 ± 0.60	45.83 ± 0.96 *
Drought	2.77 ± 0.12	7.70 ± 0.46	41.00 ± 2.10
Watered	2.75 ± 0.05	7.73 ± 0.21	37.10 ± 0.66 *

XT13: *Bacillus subtilis*; XT14: *Bacillus aryabhattai*; XT17: *Bacillus amyloliquefaciens*; XT38: *Bacillus licheniformis*; and XT110: *Bacillus aryabhattai*. Each value is a mean of three replicates ± standard deviation. * significant differences against non-inoculated control plants subjected to drought conditions according to a pairwise comparison at *p* ≤ 0.05.
